# Contributing Factors of Foodborne Illness Outbreaks — National Outbreak Reporting System, United States, 2014–2022

**DOI:** 10.15585/mmwr.ss7401a1

**Published:** 2025-03-13

**Authors:** Meghan M. Holst, Beth C. Wittry, Carolyn Crisp, Jeffrey Torres, D.J. Irving, David Nicholas

**Affiliations:** ^1^National Center for Environmental Health, CDC, Atlanta, Georgia; ^2^United States Public Health Service, Rockville, Maryland; ^3^Epidemic Intelligence Service, CDC, Atlanta, Georgia; ^4^National Center for Emerging and Zoonotic Infectious Diseases, CDC, Atlanta, Georgia; ^5^Tennessee Department of Health, Nashville, Tennessee; ^6^New York State Department of Health, Albany, New York; ^7^Department of Epidemiology & Biostatistics, School of Public Health, University at Albany, Rensselaer, New York

## Abstract

**Problem/Condition:**

Approximately 800 foodborne illness outbreaks occur in the United States each year. These outbreaks include approximately 15,000 illnesses, 800 hospitalizations, and 20 deaths. Although illnesses from outbreaks account for a small portion of all foodborne illnesses, outbreak investigations reveal how these illnesses originate by offering crucial data through epidemiologic, environmental health, and laboratory analyses and aid in outbreak mitigation and prevention.

**Period Covered:**

2014–2022.

**Description of System:**

The Foodborne Disease Outbreak Surveillance System (FDOSS), via the National Outbreak Reporting System (NORS), captures data from foodborne enteric illness outbreak investigations in the United States. Epidemiology or communicable disease control and environmental health programs of state and local health departments collect and voluntarily report the data to NORS, which is managed by CDC. These data include information about cases (e.g., case counts, symptoms, duration of illness, and health care–seeking behaviors), laboratory specimens, settings of exposure, implicated food items, and contributing factors (i.e., how the outbreak occurred). A foodborne illness outbreak is defined as two or more cases of a similar illness associated with a common exposure (e.g., shared food, venue, or experience). Data collected from an outbreak investigation help the investigator identify contributing factors to the outbreak. Contributing factors are food preparation practices, behaviors, and environmental conditions that lead to pathogens getting into food, growing in food, or surviving in food and are grouped into three categories: contamination (when pathogens and other hazards get into food), proliferation (when pathogens that are already present in food grow), and survival (when pathogens survive a process intended to kill or reduce them).

**Results:**

A total of 2,677 (40.5%) foodborne illness outbreaks reported during 2014–2022 with information on contributing factors were included in this analysis. Foodborne outbreak periods were categorized into three time frames: 2014–2016 (first), 2017–2019 (second), and 2020–2022 (third). Of the 2,677 outbreaks, 1,142 (42.7%) occurred during the first time frame, 1,130 outbreaks (42.2%) during the second time frame, and 405 outbreaks (15.1%) during the third time frame. The proportion of bacterial outbreaks increased from the first (41.9%) to the third time frame (48.4%), and the proportion of viral outbreaks decreased (33.3% to 23.2%). Over the three time frames, the proportion of outbreaks with a contamination contributing factor decreased (85.6%, 83.6%, and 81.0%, respectively). The proportion of outbreaks with a proliferation contributing factor category decreased from the first (40.3%) to the second time frame (35.0%), then increased during the third time frame (35.1%), and the proportion of outbreaks with a survival contributing factor category decreased from the first (25.7%) to the second time frame (21.9%), then increased during the third time frame (25.7%). The proportion of outbreaks with aquatic animals as an implicated food item increased from the first (12.0%) to the second time frame (18.5%), then decreased during the third time frame (18.3%). The proportion of outbreaks with land animals as an implicated food item decreased from the first (16.7%) to the second time frame (14.2%), then increased during the third time frame (15.1%).

For outbreaks with a contamination contributing factor, the proportion of food contaminated by an animal or environmental source before arriving at the point of final preparation increased over the three time frames (22.2%, 27.7%, and 32.3%, respectively), and the proportion of outbreaks with contamination from an infectious food worker through barehand contact with food decreased (20.5%, 15.2%, and 8.9%, respectively). For the proliferation category, the proportions of outbreaks associated with allowing foods to remain out of temperature control for a prolonged period during preparation and during food service or display decreased over the three time frames (15.2%, 12.2%, and 9.9%, respectively; and 13.6%, 10.4%, and 8.9%, respectively), and the proportion of improper cooling of food decreased from the first (9.4%) to the second time frame (8.8%), then increased during the third time frame (10.9%). For the survival category, the proportion of outbreaks associated with inadequate time and temperature control during initial cooking/thermal processing of food decreased from the first (12.1%) to the second time frame (9.6%) and increased during the third time frame (12.1%).

For bacterial outbreaks, cross-contamination of foods was among the top five contributing factors during the first (22.0%) and second time frames (20.8%) but not during the third time frame. Inadequate time and temperature control during initial cooking of food was among the top five contributing factors during all three time frames (23.8%, 20.4% and 20.9%, respectively). Improper cooling was not among the top five contributing factors during the first and second time frames but was during the third time frame (17.3%). For viral outbreaks, contamination from an infectious food worker through barehand contact with food was among the most common contributing factors during the first (47.1%) and second time frames (37.7%) and decreased to the third most common contributing factor during the third time frame (28.7%). Contamination from an infectious food worker through gloved-hand contact with food was among the top five contributing factors during the first (32.1%) and second time frame (25.5%) and was the most common contributing factor during the third time frame (42.5%).

**Interpretation:**

Many foodborne illness outbreaks occur because of contamination of food by an animal or environmental source before arriving at the point of final preparation. Most viral outbreaks are caused by contamination from ill food workers. The decrease in the proportion of viral outbreaks and the proportion of outbreaks with a contamination contributing factor during 2020–2022 might be attributed to effects from the COVID-19 pandemic. Nonpharmaceutical interventions (e.g., increased glove use, cleaning and disinfection, and closure of restaurant dining areas) implemented during the COVID-19 pandemic likely led to a reduction in norovirus, which is typically spread by infectious food workers. Two common contributing factors to bacterial outbreaks are allowing foods to remain out of temperature control for a prolonged period and inadequate time and temperature control during cooking. Proper time and temperature controls are needed to effectively eliminate bacterial pathogens from contaminated foods and ensure safe food operations.

**Public Health Action:**

Retail food establishments can follow science-based food safety guidelines such as the Food and Drug Administration Food Code and Hazard Analysis and Critical Control Points (HACCP) plans. Restaurant managers can mitigate contamination by ill food workers by implementing written policies concerning ill worker management, developing contingency plans for staffing during worker exclusions, and addressing reasons why employees work while sick. Health department staff members who investigate outbreaks and conduct routine inspections can encourage restaurants to follow their HACCP plans and other verified food safety practices, such as cooling, to prevent outbreaks.

## Introduction

Approximately 800 foodborne illness outbreaks occur in the United States each year. These outbreaks include approximately 15,000 illnesses, 800 hospitalizations, and 20 deaths. Illnesses from outbreaks account for a small proportion of all foodborne illnesses (*1*). Most foodborne illness outbreaks are sporadic and lack sufficient information to understand the route of exposure; however, foodborne illness outbreak investigations offer valuable epidemiologic, environmental health, and laboratory data that can help to explain how the illnesses occurred and provide information to prevent future occurrences.

Foodborne illness outbreaks in the United States are typically investigated by epidemiology or communicable disease control and environmental health programs at state and local health departments. Data from these outbreaks are reported to the Foodborne Disease Outbreak Surveillance System (FDOSS) via the National Outbreak Reporting System (NORS), which is managed by the CDC. NORS collects data on waterborne and foodborne illness outbreaks, certain fungal disease outbreaks, and enteric outbreaks transmitted by contact with environmental sources, infected persons or animals, or indeterminate or unknown modes of transmission (*2*). NORS is used to integrate and streamline surveillance; enhance state and local health department outbreak reporting; and provide needed information to CDC, health departments, and policymakers for prevention of future outbreaks. Publications using FDOSS data have provided insights on common pathogen-food pairings and investigation details on unique pathogen outbreaks to explain how the outbreaks occurred (*3*).

As part of an outbreak investigation, an environmental assessment is an observation of the retail food establishment where the outbreak occurred that identifies factors contributing to the outbreak. Contributing factors are food preparation practices, behaviors, and environmental conditions that lead to pathogens getting into food, growing in food, or surviving in food and are reported to FDOSS along with other foodborne illness outbreak data. Contributing factors are identified after an investigation is complete and the epidemiologic, laboratory, and environmental health data are reviewed. An environmental assessment uses epidemiologic data to target investigation activities (e.g., observations of the kitchen, interviews with food workers and managers, and a review of records) to determine how the outbreak occurred by identifying contributing factors (*4*). Food safety experts from Food Safety Consultation and Training, the New York Department of Health, and Health Canada developed the concept and definitions of contributing factors and grouped them into three categories: contamination (when pathogens and other hazards get into food), proliferation (when pathogens that are already present in food grow), and survival (when pathogens survive a process intended to kill or reduce them) (*5*). CDC, the Food and Drug Administration (FDA), and state health departments revised these contributing factors to address changes in culinary practices and to encompass the farm-to-fork continuum and demonstrate how foodborne illness outbreaks occur (*6*). One example of a contributing factor scenario is when food is held at an improper temperature for a long time, allowing bacteria to grow. An outbreak can then occur when persons eat this food. Bacterial proliferation from inadequate holding temperature resulting from an improper practice would likely be a contributing factor of this outbreak.

CDC has previously reported contributing factors and etiologies of outbreaks. For example, analyses have found norovirus outbreaks often are associated with ill food workers contaminating food, and *Salmonella* outbreaks are associated with cross-contamination. These insights help investigators understand how outbreaks occur (*7*). Identifying outbreak contributing factors provides mechanisms to guide the implementation of effective control measures and supports food safety research. For example, FDA used contributing factor data to guide the development of their Retail Food Risk Factor study, which described the frequency of practices that can lead to foodborne illness (i.e., foodborne illness risk factors) in retail establishments. The findings have informed retail food safety initiatives and intervention strategies (*8*).

CDC has not summarized and published descriptive data on outbreak contributing factors since 2016. Disseminating this information is crucial for understanding and preventing foodborne illness outbreaks. New cooking trends, policy changes, and the COVID-19 pandemic might have influenced the frequency of these contributing factors. For example, recent trends of drinking unpasteurized milk and eating undercooked chicken livers likely influence the pattern of outbreak contributing factors identified (*9*,*10*). This analysis describes patterns in outbreak contributing factor data over time and during the COVID-19 pandemic and examines the differences in outbreak contributing factors between bacterial and viral outbreaks. Health departments and retail food establishment owners and employees can use this information to understand common reasons why foodborne outbreaks occur and implement effective food safety policies and practices and, if needed, corrective public health actions.

## Methods

### Description of the System, Data Collection, and Case Definition

FDOSS captures data from foodborne disease outbreak investigations in the United States. Epidemiology or communicable disease control and environmental health programs at state and local health departments conduct outbreak investigations, collect and enter data into NORS, and voluntarily report to FDOSS, which is managed by CDC (*2*).

### Variables

FDOSS data include information about outbreak illness cases (e.g., case counts, symptoms, duration of illness, and health care–seeking behaviors), laboratory specimens, settings of exposure, food items implicated in the outbreak, and contributing factors (i.e., how the outbreak occurred). Health department staff members review all available data and determine the contributing factors of the outbreak. More than one contributing factor can be identified for an outbreak. 

Year is based on when the first primary case reported their illness onset. For this analysis, outbreak period was categorized into three time frames: 2014–2016 (first), 2017–2019 (second), and 2020–2022 (third). The preset list of 30 contributing factors was modified in the NORS system in 2022 to improve understanding and identification during an outbreak investigation. The previous years’ contributing factors were matched in NORS to the revised contributing factors. The etiologic agents were placed into five categories: 1) bacterial (*Salmonella*, *Campylobacter*, *Shigella*, *Clostridium perfringens*, *Escherichia coli*, *Bacillus cereus*, *Staphylococcus aureus*, *Vibrio*, *Brucella*, *Listeria*, *Streptococcus*, and other bacterium), 2) chemical and toxin (scombroid toxin, ciguatoxin, mycotoxin, neurotoxic and paralytic shellfish poison, plant or herbal toxin, cleaning agent, and other chemical or toxin), 3) parasitic (*Cryptosporidium*, *Trichinella*, *Giardia*, *Cyclospora*, *Toxoplasma*, and other parasite), 4) viral (norovirus, sapovirus, hepatitis A virus, rotavirus, and other virus), and 5) unknown. Two bacterial or viral pathogens could be reported for an outbreak; as a result, multiple confirmed or suspected etiologic agents could be attributed to one outbreak. However, a pathogen, chemical, or toxin could not be assigned to more than one etiologic category. A confirmed etiology is often based on laboratory confirmation and suspected etiology is often based on a combination of laboratory, epidemiologic, clinical, or other evidence. Outbreaks with unknown etiologic agents were also included in the analysis. 

Where the food was prepared and eaten was categorized as a restaurant (sit-down, fast-food, buffet, or other restaurant), catering or banquet facility, nonpublic setting (private home, picnic, potluck, or other nonpublic setting), institutional location (school, prison, office, hospital, or nursing home), other commercial location (grocery store or farm), other location, or unknown. The food items implicated in the outbreak were categorized according to the Interagency Food Safety Analytics Collaboration scheme. The categories include aquatic animals (fish, shellfish, and other aquatic animals), land animals (dairy, game, meat, poultry, and eggs), plants (oils, sugars, produce, grains, beans, nut, and seeds), multiple items, and other items (*11*).

### Data Analysis

To characterize contributing factors for outbreaks of viral and bacterial foodborne illness and to examine how these factors varied before and during the COVID-19 pandemic, CDC analyzed 2014–2022 FDOSS data. Prior to obtaining data from NORS, NORS staff collaborated with health departments to clean the data by rectifying misaligned dates and reconciling discrepancies. Descriptive analyses were conducted for the variables included in the analysis. Frequencies for contributing factors were calculated over the three time frames, and the top five contributing factors are presented by time frame for the two largest categories of outbreak type (bacterial and viral). Data cleaning and analyses were conducted using SAS (version 9.4; SAS Institute) and Microsoft Excel. This activity was reviewed by CDC, deemed not research, and was conducted consistent with applicable Federal law and CDC policy.*

## Results

During 2014–2022, a total of 6,618 foodborne illness outbreaks were reported to NORS. Outbreaks were excluded from analysis if a contributing factor was not reported (n = 3,788 [57.2%]) or if a pathogen was identified in more than one etiologic category (n = 23 [0.3%]). An additional 126 (1.9%) viral outbreaks were excluded because a contributing factor that was not biologically feasible was reported. Viruses are initially spread by a contamination event, and they can further contaminate establishment surfaces if not properly addressed. Viruses do not multiply outside the human body so proliferation and survival contributing factors do not apply to these outbreaks (*12*). Lastly, four (<0.1%) outbreaks were excluded because the contributing factors for the outbreaks were not plausible; these included raw milk outbreaks with survival contributing factor (n = 3) and a scombroid outbreak with hot-holding contributing factor (n = 1). The final dataset consisted of 2,677 foodborne illness outbreaks. 

### Outbreak Characteristics

Of the 2,677 outbreaks included during three time frames (2014–2022), a total of 1,142 (42.7%) occurred during 2014–2016 (first time frame), 1,130 (42.2%) during 2017–2019 (second), and 405 (15.1%) during 2020–2022 (third) (Table 1). The proportion of bacterial outbreaks increased from the first to the third time frame (41.9% to 48.4%), and the proportion of viral outbreaks decreased (33.3% to 23.2%). The proportion of outbreaks associated with a contamination contributing factor decreased over the three time frames (85.6%, 83.6%, and 81.0%, respectively). The proportion of outbreaks associated with a proliferation contributing factor category decreased from the first (40.3%) to the second time frame (35.0%), then remained constant during the third time frame (35.1%), and the survival contributing factor category decreased from the first (25.7%) to the second time frame (21.9%), then returned to the first time frame proportion (25.7%). The proportion of outbreaks associated with implicated aquatic animal food items increased from the first (12.0%) to the second time frame (18.5%) and decreased slightly during the third time frame (18.3%), and the proportion of outbreaks associated with implicated land animal food items decreased from the first (16.7%) to the second time frame (14.2%), then increased during the third time frame (15.1%) (Table 1).

**TABLE 1:** Number of foodborne illness outbreaks, by selected characteristics and time frame — Foodborne Disease Outbreak Surveillance System, United States, 2014–2022*

Characteristic	2014–2016 (n = 1,142)	2017–2019 (n = 1,130)	2020–2022 (n = 405)	Total (n = 2,677)
	No. of outbreaks (%)			
**Etiologic agent identified**				
Bacterial	478 (41.9)	471 (41.7)	196 (48.4)	**1,145 (42.8)**
Chemical and toxin	101 (8.8)	125 (11.1)	51 (12.6)	**277 (10.3)**
Parasitic	15 (1.3)	59 (5.2)	18 (4.4)	**92 (3.4)**
Viral	380 (33.3)	345 (30.5)	94 (23.2)	**819 (30.6)**
Unknown	168 (14.7)	130 (11.5)	46 (11.4)	**344 (12.9)**
**Etiologic agent^†^**				
Confirmed	732 (64.1)	721 (63.8)	263 (64.9)	**1,716 (64.1)**
Suspected	303 (26.5)	385 (34.1)	148 (36.5)	**836 (31.2)**
Unknown pathogen, chemical, or toxin	168 (14.7)	130 (11.5)	46 (11.4)	**344 (12.9)**
**No. of primary cases**				
2–10	678 (59.4)	697 (61.7)	247 (61.0)	**1,622 (60.6)**
11–20	207 (18.1)	189 (16.7)	69 (17.0)	**465 (17.4)**
21–30	87 (7.6)	86 (7.6)	34 (8.4)	**207 (7.7)**
≥31	170 (14.9)	158 (14.0)	55 (13.6)	**383 (14.3)**
**Contributing factor category^†^**				
Contamination	977 (85.6)	945 (83.6)	328 (81.0)	**2,250 (84.0)**
Proliferation	460 (40.3)	395 (35.0)	142 (35.1)	**997 (37.2)**
Survival	293 (25.7)	248 (21.9)	104 (25.7)	**645 (24.1)**
**Implicated food category**				
Aquatic animals	137 (12.0)	209 (18.5)	74 (18.3)	**420 (15.7)**
Land animals	191 (16.7)	160 (14.2)	61 (15.1)	**412 (15.4)**
Plants	96 (8.4)	119 (10.5)	46 (11.4)	**261 (9.7)**
Multiple	246 (21.5)	198 (17.5)	81 (20.0)	**525 (19.6)**
Other	13 (1.1)	17 (1.5)	9 (2.2)	**39 (1.5)**
No implicated food item	459 (40.2)	427 (37.8)	134 (33.1)	**1,020 (38.1)**
**Where the food was prepared^†^**				
Restaurant (sit-down, fast-food, buffet, or other)	658 (57.6)	714 (63.2)	238 (58.8)	**1,610 (60.1)**
Catering or banquet facility	146 (12.8)	135 (11.9)	36 (8.9)	**317 (11.8)**
Nonpublic setting	176 (15.4)	184 (16.3)	72 (17.8)	**432 (16.1)**
Institutional location (school, prison, office, hospital, or nursing home)	73 (6.4)	60 (5.3)	39 (9.6)	**172 (6.4)**
Commercial location (grocery store or farm)	104 (9.1)	84 (7.4)	43 (10.6)	**231 (8.6)**
Other	7 (0.6)	8 (0.7)	1 (0.2)	**16 (0.6)**
Unknown	11 (1.0)	14 (1.2)	5 (1.2)	**30 (1.1)**
**Where the food was eaten^†^**				
Restaurant (sit-down, fast-food, buffet, or other)	589 (51.6)	643 (56.9)	198 (48.9)	**1,430 (53.4)**
Catering or banquet facility	89 (7.8)	70 (6.2)	16 (4.0)	**175 (6.5)**
Nonpublic setting	246 (21.5)	243 (21.5)	103 (25.4)	**592 (22.1)**
Institutional location (school, prison, office, hospital, or nursing home)	162 (14.2)	159 (14.1)	79 (19.5)	**400 (14.9)**
Commercial location (grocery store or farm)	48 (4.2)	43 (3.8)	17 (4.2)	**108 (4.0)**
Other	60 (5.3)	63 (5.6)	21 (5.2)	**144 (5.4)**
Unknown	10 (0.9)	21 (1.9)	14 (3.5)	**45 (1.7)**

* N = 2,677 reported outbreaks with a contributing factor identified. More than one contributing factor could be identified for an outbreak.

^†^ Categories are not mutually exclusive and totals can sum to >100%.

The proportion of outbreaks with implicated food that was prepared and eaten at restaurants increased from the first to the second time frame; however, the proportions decreased from the second to the third time frames (prepared at restaurant: 57.6%, 63.2%, and 58.8%, respectively; eaten at restaurant: 51.6%, 56.9%, and 48.9%, respectively). The proportion of outbreaks with institutional and commercial food preparation locations decreased from the first to the second time frame, then increased during the third time frame (prepared at institutional location: 6.4%, 5.3%, and 9.6%, respectively; prepared at commercial location: 9.1%, 7.4%, and 10.6%, respectively). The proportion of outbreaks in which the implicated food was eaten at institutional locations was similar during the first (14.2%) and second time frame (14.1%), then increased during the third time frame (19.5%). The proportion of outbreaks of which the implicated food was eaten at commercial locations decreased from the first (4.2%) to the second time frame (3.8%), then returned to the first time frame proportion (4.2%) (Table 1).

### Contributing Factors 

Overall, food contaminated by an animal or environmental source before arriving at the point of final preparation (26.0%) was the most common contributing factor and increased over the three time frames (22.2%, 27.7%, and 32.3%, respectively) (Table 2). Contamination from an infectious food worker through barehand contact with food (16.5%) was the second most common contributing factor overall and during all three time frames (20.5%, 15.2%, and 8.9%, respectively). Other common contributing factors were the contamination contributing factor of contamination from an infectious food worker through unknown hand contact with food or indirect contact with food (13.1%) and the proliferation contributing factor of allowing foods to remain out of temperature control for a prolonged period during preparation (13.1%) (Table 2).

**TABLE 2:** Number of foodborne illness outbreaks, by contributing factor and time frame — Foodborne Disease Outbreak Surveillance System, United States, 2014–2022*

Contributing factor	2014–2016 (n = 1,142)	2017–2019 (n = 1,130)	2020–2022 (n = 405)	Total (n = 2,677)
	No. (%)			
**Contamination**				
Food contaminated by animal or environmental source before arriving at point of final preparation	253 (22.2)	313 (27.7)	131 (32.3)	**697 (26.0)**
Contamination from infectious food worker through barehand contact with food	234 (20.5)	172 (15.2)	36 (8.9)	**442 (16.5)**
Contamination from infectious food worker through unknown hand contact with food or indirect contact with food	151 (13.2)	148 (13.1)	51 (12.6)	**350 (13.1)**
Cross-contamination of foods	155 (13.6)	131 (11.6)	36 (8.9)	**322 (12.0)**
Contamination from infectious food worker through gloved-hand contact with food	150 (13.1)	111 (9.8)	48 (11.9)	**309 (11.5)**
Other	143 (12.5)	128 (11.3)	30 (7.4)	**301 (11.2)**
Toxin or chemical agent naturally part of tissue in food	108 (9.5)	130 (11.5)	47 (11.6)	**285 (10.6)**
Food contaminated by animal or environmental source at point of final preparation/sale	85 (7.4)	48 (4.2)	13 (3.2)	**146 (5.5)**
Contamination from infectious nonfood worker through direct or indirect contact with food	51 (4.5)	35 (3.1)	14 (3.5)	**100 (3.7)**
Poisonous substance accidentally added to food	7 (0.6)	4 (0.4)	6 (1.5)	**17 (0.6)**
Ingredients toxic in large amounts accidentally added to food	4 (0.4)	3 (0.3)	0 (—)	**7 (0.3)**
Container or equipment used to hold or convey food was made with toxic substances	1 (0.1)	2 (0.2)	0 (—)	**3 (0.1)**
Poisonous substances or infectious agent intentionally added to food to cause illness	2 (0.2)	0 (—)	1 (0.2)	**3 (0.1)**
**Proliferation**				
Allowing foods to remain out of temperature control for a prolonged period during preparation	174 (15.2)	138 (12.2)	40 (9.9)	**352 (13.1)**
Allowing foods to remain out of temperature control for a prolonged period during food service or display	155 (13.6)	118 (10.4)	36 (8.9)	**309 (11.5)**
Inadequate cold holding temperature due to an improper practice	128 (11.2)	107 (9.5)	37 (9.1)	**272 (10.2)**
Inadequate hot holding temperature due to an improper practice	124 (10.9)	111 (9.8)	36 (8.9)	**271 (10.1)**
Improper cooling of food	107 (9.4)	100 (8.8)	44 (10.9)	**251 (9.4)**
Inadequate cold holding temperature due to malfunctioning refrigeration equipment	62 (5.4)	68 (6.0)	21 (5.2)	**151 (5.6)**
Other	56 (4.9)	48 (4.2)	26 (6.4)	**130 (4.9)**
Inadequate hot holding temperature due to malfunctioning equipment	16 (1.4)	16 (1.4)	7 (1.7)	**39 (1.5)**
Inadequate non-temperature–dependent processes	9 (0.8)	5 (0.4)	2 (0.5)	**16 (0.6)**
Extended refrigeration of food for an unsafe amount of time	6 (0.5)	4 (0.4)	1 (0.2)	**11 (0.4)**
Inadequate reduced oxygen packaging of food	4 (0.4)	0 (—)	0 (—)	**4 (0.1)**
**Survival**				
Inadequate time and temperature control during initial cooking/thermal processing of food	138 (12.1)	108 (9.6)	49 (12.1)	**295 (11.0)**
Inadequate time and temperature control during reheating of food	88 (7.7)	82 (7.3)	26 (6.4)	**196 (7.3)**
Other	82 (7.2)	83 (7.3)	26 (6.4)	**191 (7.1)**
Inadequate non–temperature-dependent process	25 (2.2)	12 (1.1)	5 (1.2)	**42 (1.6)**
Inadequate time and temperature control during freezing of food designed for pathogen description	11 (1.0)	5 (0.4)	2 (0.5)	**18 (0.7)**
No attempt was made to inactivate the contaminant through initial cooking/thermal processing, freezing, or chemical processes	0 (—)	0 (—)	1 (0.2)	**1 (—)**

* N = 2,677 reported outbreaks with a contributing factor identified. More than one contributing factor could be identified for an outbreak.

Overall, the most common proliferation contributing factors were foods remaining out of temperature control for a prolonged period during preparation (13.1%) and during food service or display (11.5%). Both decreased over the three time frames (15.2%, 12.2%, and 9.9%, respectively; 13.6%, 10.4%, and 8.9%, respectively). Overall, the most common survival contributing factors were inadequate time and temperature control during initial cooking or thermal processing of food (11.0%) and during reheating of food (7.3%) (Table 2).

### Bacterial and Viral Outbreaks

Contamination of food by an animal or environmental source before arriving at the point of final preparation was the most common contributing factor of bacterial outbreaks across all three time frames (41.8%, 42.5%, and 50.5%, respectively) (Table 3). Cross-contamination of foods was one of the top five contributing factors for bacterial outbreaks for the first (22.0%) and second time frame (20.8%) but not for the third time frame. Inadequate time and temperature control during initial cooking of food was among the top five contributing factors during all three time frames (23.8%, 20.4%, and 20.9%, respectively). Allowing foods to remain out of temperature control for a prolonged period during preparation was among the top five contributing factors during all three time frames (30.1%, 22.7%, and 15.3%, respectively). Improper cooling did not appear among the top five contributing factors for bacterial outbreaks for the first and second time frames but did appear during the third time frame (17.3%) (Table 3).

**TABLE 3:** Number of foodborne illness outbreaks, by top five contributing factors for bacterial and viral outbreaks and time frame — Foodborne Disease Outbreak Surveillance System, United States, 2014–2022*

Bacterial					
2014–2016 (n = 478)		2017–2019 (n = 471)		2020–2022 (n = 196)	
Contributing factor	No. (%)	Contributing factor	No. (%)	Contributing factor	No. (%)
Food contaminated by animal or environmental source before arriving at point of final preparation	200 (41.8)	Food contaminated by animal or environmental source before arriving at point of final preparation	200 (42.5)	Food contaminated by animal or environmental source before arriving at point of final preparation	99 (50.5)
Allowing foods to remain out of temperature control for a prolonged period during preparation	144 (30.1)	Allowing foods to remain out of temperature control for a prolonged period during preparation	107 (22.7)	Inadequate time and temperature control during initial cooking of food	41 (20.9)
Inadequate time and temperature control during initial cooking of food	114 (23.8)	Cross-contamination of foods	98 (20.8)	Improper cooling of food	34 (17.3)
Allowing foods to remain out of temperature control for a prolonged period during food service or display	111 (23.2)	Inadequate time and temperature control during initial cooking of food	96 (20.4)	Allowing foods to remain out of temperature control for a prolonged period during preparation	30 (15.3)
Cross-contamination of foods	105 (22.0)	Inadequate hot holding temperature due to an improper practice	87 (18.5)	Allowing foods to remain out of temperature control for a prolonged period during food service or display	28 (14.3)
**Viral^†^**					
**2014–2016 (n = 380)**		**2017–2019 (n = 345)**		**2020–2022 (n = 94)**	
**Contributing factor**	**No. (%)**	**Contributing factor**	**No. (%)**	**Contributing factor**	**No. (%)**
Contamination from infectious food worker through barehand contact with food	179 (47.1)	Contamination from infectious food worker through barehand contact with food	130 (37.7)	Contamination from infectious food worker through gloved-hand contact with food	40 (42.6)
Contamination from infectious food worker through gloved-hand contact with food	122 (32.1)	Contamination from infectious food worker through unknown hand contact	129 (37.4)	Contamination from infectious food worker through unknown hand contact	38 (40.4)
Contamination from infectious food worker through unknown hand contact	114 (30.0)	Contamination from infectious food worker through gloved-hand contact with food	88 (25.5)	Contamination from infectious food worker through barehand contact with food	27 (28.7)
Other source of contamination	45 (11.8)	Other source of contamination	43 (12.5)	Contamination from infectious nonfood worker through direct or indirect contact with food	11 (11.7)
Contamination from infectious nonfood worker through direct or indirect contact with food	37 (9.7)	Food contaminated by animal or environmental source before arriving at point of final preparation	34 (9.8)	Food contaminated by animal or environmental source before arriving at point of final preparation	6 (6.4)

* N = 2,677 reported outbreaks with a contributing factor identified. More than one contributing factor could be identified for an outbreak.

^†^ Proliferation and survival factors do not apply to these outbreaks.

Contamination from an infectious food worker through barehand contact with food was the most common contributing factor of viral outbreaks during the first (47.1%) and second time frames (37.7%) but decreased to the third most common contributing factor during the third time frame (28.7%). Contamination from an infectious food worker through gloved-hand contact with food was one of the top five contributing factors for viral outbreaks for the first (32.1%) and second time frames (25.5%) and was the most common contributing factor during the third time frame (42.6%). Contamination from an infectious nonfood worker through direct or indirect contact with food was one of the top five contributing factors for viral outbreaks for the first time frame (9.7%), did not appear during the second time frame, then appeared as the fourth most common during the third time frame (11.7%) (Table 3).

## Discussion

This report is the most recent to examine contributing factors of foodborne illness outbreaks reported to NORS. During 2014–2022, the most common contributing factor to outbreaks was food contaminated by an animal or environmental source before arriving at the point of final preparation, which can occur pre- or post-harvest. Certain foods contaminated pre- or post-harvest are intended to be consumed raw (e.g., leafy greens and fresh produce), and controls (e.g., sanitation controls at a processing facility) are required before these foods reach the retail establishment to mitigate contamination (*13*,*14*). Certain system failures still allow pathogens to contaminate foods that should be free of pathogens, but these controls are beyond the scope of this report. Cooking standards (e.g., ensuring raw chicken is cooked to an internal temperature of 165°F [73.9°C]) are established for foods intended to be cooked, and retail food establishments can further safeguard against this kind of contamination by ensuring thorough cooking processes, validated through Hazard Analysis and Critical Control Points (HACCP) plans, to eliminate bacterial pathogens (*15*). Health department staff members who investigate outbreaks and conduct routine inspections can encourage food workers to follow HACCP plans and explain the importance of using the plans to prepare safe food.

For bacterial outbreaks, improper cooling of food was the third most common contributing factor during the third time frame; however, it did not appear among the top five contributing factors during the first two time frames. During the previous decade, researchers have conducted various studies on cooling practices; these studies guide educational activities on best cooling practices for food inspectors and outbreak investigators (*16**–**20*). One study used modeling techniques to identify optimal food cooling practices for different food types and container depths (*19*). Investigators can apply this research to determine how establishments manage food cooling operations. The development of research on proper cooling techniques might suggest that investigators are better equipped to recognize when improper cooling occurs. Cooling is a complex process, and health department staff who investigate outbreaks and conduct routine inspections can validate cooling practices at the establishment by reviewing cooling logs, ensuring there is a working food thermometer, discussing cooling practices with food workers, and if possible, observing food cooling.

A study on contributing factors during 2006–2007 indicated improper time and temperature procedures were the main causes of restaurant-associated foodborne illness outbreaks (*21*). The study identified similar proliferation factors for bacterial outbreaks, such as allowing foods to remain out of temperature control for a prolonged period and inadequate time and temperature control during cooking. Findings from previous studies and this analysis indicate that inadequate food temperature is a consistent and persistent contributing factor to outbreaks (*8*).

Ill food workers, through barehand, gloved-hand, or unknown contact, play a large role in food contamination and are a consistent cause of foodborne illness outbreaks (*21*). The proportion of outbreaks attributed to barehand contact with food from an infectious worker stayed consistent during the first two time frames and then decreased during the third time frame. The decrease of outbreaks attributed to barehand contact is likely a result of nonpharmaceutical interventions used during the COVID-19 pandemic. Nonpharmaceutical interventions (e.g., increased glove use, enhanced cleaning and disinfection, and the closure of restaurant dining areas) implemented during the COVID-19 pandemic may have contributed to a reduction in norovirus transmission, which is typically spread by infectious food workers (*22**–**24*). Previous studies have demonstrated that adherence to rules regarding the exclusion of ill workers from workplaces and proper hand hygiene has the greatest impact on reducing worker and consumer illnesses (*25*). However, many barriers to excluding ill food workers exist, such as staffing shortages and potential job or income loss (*26*,*27*). Restaurant managers can mitigate these risks by implementing written policies, developing contingency plans for staffing during worker exclusions, and addressing reasons why employees work while ill (*28*).

The 64.5% decrease in outbreaks reported to NORS from 2014–2016 (first time frame) to 2020–2022 (third time frame) is likely a result of staffing and resource limitations at state and local health departments that prevented outbreak investigation during the COVID-19 pandemic. The decrease also might be explained by persons choosing to eat at home instead of a public setting and restaurant closures to minimize the spread of COVID-19 (*22**,**29**,**30*). CDC recommendations during the pandemic discouraged large events (e.g., weddings), resulting in fewer outbreaks associated with food prepared and eaten at catering and banquet facilities (*31*). However, outbreaks where food was prepared and eaten at an institutional location (e.g., school, prison, office, hospital, and nursing home) increased during the pandemic. Many of these settings are residential or provide temporary housing, so meal services needed to continue throughout the pandemic. In these settings, large quantities of food were likely prepared, then the meals were either brought to rooms for social distancing or held for longer times to allow for more group mealtimes to social distance in a communal cafeteria (*32*). These prolonged holding periods could explain inadequate food temperature contributing factors observed during the pandemic. In addition, staff members might not have wanted to work during the pandemic because of a risk for contracting COVID-19 or were absent because of lengthy quarantine requirements (*22**,**33**,**34*). When a staffing shortage exists, restaurants and food service establishments can consider modifying operations, such as limiting the menu and simplifying the food operations and processes. Modifying operations might reduce the workload on employees so they do not miss steps in a food preparation processes (e.g., not complete all steps during the cooling process or wash hands properly) that could affect the safety of food they serve.

Cross-contamination, which occurs when raw animal foods, such as poultry, meat, and seafood contaminate ready-to-eat products, was a common contributing factor during the first two time frames but not during the third time frame (*35*). During the COVID-19 pandemic, retail food establishments intensified cleaning and disinfection of frequently touched surfaces, shared tools, and equipment, and emphasized handwashing to mitigate the spread of COVID-19 (*22*). These measures likely played a role in indirectly reducing the transmission of pathogens through cross-contamination.

Most of the outbreaks examined occurred in settings where food safety considerations apply; however, approximately 16.5% of outbreaks during each time frame included a food item that was prepared in nonpublic settings to include private homes, but also home food preparation for events such as picnics, potlucks, and celebrations. Many food safety considerations for retail settings, such as HACCP plans and ill worker policies, might not apply to the average person who prepares food; however, the concepts behind these considerations are important. Food safety information from CDC, FDA, and the U.S. Department of Agriculture is available (https://www.foodsafety.gov/), providing information in plain language, considerations for special populations (e.g., pregnant women, persons aged ≥65 years, and those with immunocompromise), and food recalls.

## Limitations

The findings in this report are subject to at least four limitations. First, because NORS operates as a voluntary reporting system, the data might not represent all outbreaks and illnesses. Second, reporting practices vary among health departments because of the level of outbreak response training and adequate staffing. These differences can affect accurate contributing factor reporting and the ability of public health agencies to properly identify and investigate foodborne outbreaks. Third, the findings of these analyses might differ slightly from previous or future reports because state health departments can submit, update, or delete reports in the NORS system at any time. Finally, although the definitions of contributing factors were adjusted in 2022, the underlying concepts remain consistent; however, misclassification might occur depending on the circumstances of the outbreak.

## Future Directions

 Areas for improved surveillance of foodborne illness outbreaks include determining the effect of interventions and achieving more complete data collection. Longitudinal studies that track the effectiveness of interventions could characterize food safety mitigation strategies that prevent outbreaks over extended periods. In addition, a need exists to explore why investigators do not always identify contributing factors to foodborne outbreaks. Analyzing outbreak data in which the cause of the outbreak was identified can elucidate why other investigators might not be able to identify a contributing factor. The training of public health staff members in conducting foodborne outbreak investigations also is crucial. By evaluating the current training programs and identifying gaps or areas for improvement, future research can help ensure that public health professionals are adequately equipped to manage outbreaks more effectively, ultimately enhancing food safety and public health outcomes. Investigation skills are particularly important during and after a pandemic when food trends are changing and staff turnover is high. Finally, this analysis can be repeated in future years with new data to understand how contributing factors might change over time, including whether changes seen during the COVID-19 pandemic persist.

## Conclusion

Contamination from infectious food workers can be managed by implementing a staffing plan to exclude ill food workers and addressing why employees work while ill (e.g., do not want fellow employees to be short staffed and cannot sacrifice their pay). Inadequate food temperatures continue to be a common cause of bacterial outbreaks and can be controlled by using written procedures and verified HACCP plans. Health department staff members who investigate outbreaks and conduct routine inspections can encourage restaurants to follow their HACCP plans and other verified food safety practices, such as proper cooling, to prevent outbreaks. Inadequate food temperatures might have been exacerbated by the COVID-19 pandemic when staff member turnover and absenteeism was high, resulting in new, untrained, or overextended food workers engaging in unsafe food practices (*22*). Federal and state government organizations can consider developing pandemic preparedness plans for safe food operations (*36*). These plans can address different levels of the food supply chain, from preharvest to the retail establishment, and lessons learned from the COVID-19 pandemic, such as how to manage ill food workers and how to prevent improper food temperatures. Finally, outbreak investigators should identify contributing factors when possible to better understand outbreak etiologies. Identifying contributing factors relies heavily on a robust environmental health and food safety program at health departments that can conduct timely and comprehensive environmental assessments (*37*). Understanding the national outbreak landscape can direct public health guidance and policies to influence national food safety training curriculums.

## Footnotes

* 45 C.F.R. part 46.102(l)(2), 21 C.F.R. part 56; 42 U.S.C. Sect. 241(d); 5 U.S.C. Sect. 552a; 44 U.S.C. Sect. 3501 et seq.
